# Concurrent brain structural and functional alterations in patients with migraine without aura: an fMRI study

**DOI:** 10.1186/s10194-020-01203-5

**Published:** 2020-12-07

**Authors:** Zhengjie Li, Jun Zhou, Lei Lan, Shirui Cheng, Ruirui Sun, Qiyong Gong, Max Wintermark, Fang Zeng, Fanrong Liang

**Affiliations:** 1 Acupuncture & Tuina School / The 3rd Teaching Hospital, Chengdu University of Traditional Chinese Medicine, Chengdu, 610036 Sichuan China; 2 Radiology Department, Stanford University, Stanford, California 94305 USA; 3 Huaxi MR Research Center, West China Hospital of Sichuan University, Chengdu, 610041 Sichuan China

**Keywords:** Migraine, Functional magnetic resonance imaging (fMRI), Regional homogeneity (Reho), Voxel-based Morphometry (VBM), Functional connectivity (FC), Median raphe nuclei (MRN)

## Abstract

**Objectives:**

To explore the possible concurrent brain functional and structural alterations in patients with migraine without aura (MwoA) patients compared to healthy subjects (HS).

**Methods:**

Seventy-two MwoA patients and forty-six HS were recruited. 3D-T1 and resting state fMRI data were collected during the interictal period for MwoA and HS. Voxel-based morphometry (VBM) for structure analysis and regional homogeneity (Reho) for fMRI analysis were applied. The VBM and Reho maps were overlapped to determine a possible brain region with concurrent functional and structural alteration in MwoA patients. Further analysis of resting state functional connectivity (FC) alteration was applied with this brain region as the seed.

**Results:**

Compared with HS, MwoA patients showed decreased volume in the bilateral superior and inferior colliculus, periaqueductal gray matter (PAG), locus ceruleus, median raphe nuclei (MRN) and dorsal pons medulla junction. MwoA patients showed decreased Reho values in the middle occipital gyrus and inferior occipital gyrus, and increased Reho values in the MRN. Only a region in the MRN showed both structural and functional alteration in MwoA patients. Pearson correlation analysis showed that there was no association between volume or Reho values of the MRN and headache frequency, headache intensity, disease duration, self-rating anxiety scale or self-rating depression scale in MwoA patients. Resting state functional connectivity (FC) with the MRN as the seed showed that MwoA patients had increased FC between the MRN and PAG.

**Conclusions:**

MRN are involved in the pathophysiology of migraine during the interictal period. This study may help to better understand the migraine symptoms.

**Trial registration:**

NCT01152632. Registered 27 June 2010.

## Introduction

Migraine is a paroxysmal neurological disorder, classically characterized by unilateral throbbing, pulsating headache associated with nausea, vomiting, photophobia, phonophobia, or allodynia [1]. Between migraine attacks, migraineurs often have accompanying symptoms, such as fatigue, sleep disturbances [2], altered cognition and/or mood changes [3]. According to the 3rd edition of International Classification of Headache Disorders (the most commonly used criteria in research), there are two major types of migraine: migraine without aura (MwoA) and migraine with aura [4]. About 64% of migraine patients fall into MwoA subtype, which is the most prevalent type among migraineurs [5]. Migraine has become an important public health and social issue due to its high prevalence worldwide [6], large medical burden [7], disabling effects [7], and serious reduction in quality of life [8]. However, the pathophysiology of migraine is not fully understood.

Because migraine is mainly a disorder of the brain, neuroimaging studies have great potential to provide insight into the pathophysiology of migraine. Studies using neuroimaging techniques to evaluate brain function or structure in migraine have reported with increasing frequency in recent decades. However, many of these studies either focus on structural or functional changes [9–13], rather than including both. Furthermore, most of these studies are difficult to replicate, and no reproducible biomarkers of migraine have been identified [13]. The significance of defining concurrent functional and structural differences may provide specific insights into the unfolding brain’s adaptive or maladaptive changes in migraineurs.

There are some previous works that have used both structural and functional MRI in migraine. Some studies reporting concurrent brain functional and structural differences in migraineurs are focusing on particular pre-defined brain regions (such as cerebellum [14], hippocampus [15], or only cortical regions [16]) or combining structural analysis with hypothesis-driven functional analysis methods [17–19]. One recent study combined global structural analysis (tensor-based morphometry) and hypothesis-free functional analysis (independent component analysis) in migraineurs [20]. In this study, we aim to combine whole brain data-driven analysis methods, i.e. voxel-based morphometry (VBM) [21] for structural analysis, and regional homogeneity (Reho) [22] for functional analysis, in order to explore the possible concurrent functional and structural alterations in migraineurs relative to healthy subjects.

## Methods

This study represents the baseline assessment of a registered clinical trial listed on clinicaltrials.gov (NCT01152632, June 27, 2010). The study protocol was approved by the ethics committee of the first teaching hospital of Chengdu University of Traditional Chinese Medicine. Participants were enrolled from the outpatient department of the 3rd Teaching Hospital and the campus of Chengdu University of Traditional Chinese Medicine.

### Participants

Seventy-two migraineurs without aura (MwoA) were recruited. These patients were diagnosed according to the International Classification of Headache Disorders, 2nd Edition ICHD-II MwoA criteria [23]. Inclusion criteria required all subjects (1) being between 17 and 45 years of age and right-handed; (2) not taking any prophylactic headache medicine, or acupuncture treatment within the last 3 months before the recruitment; (3) migraines of at least 6 months duration; (4) at least one headache attack per month during the last 3 months, and (5) having signed a written consent form. Subjects were excluded if they had any history of (1) alcohol or drug abuse; (2) pregnancy or experiencing lactation; (3) suffering from psychiatric, neurologic, cardiovascular, respiratory or renal illnesses; (4) suffering from any other chronic pain conditions or having a history of head trauma, with loss of consciousness; (5) having fMRI contraindications, such as claustrophobia.

Forty-six age-matched, right-handed healthy subjects (HS), free from any chronic pain condition (such as migraine and tension type headache), were recruited for this study as controls. In order to exclude organic disease carriers, a basic evaluation was performed in each subject before recruitment, including a review of their medical history, physical examination, hepatic and renal function tests, and routine analysis of blood, urine, and stool. Individuals with any abnormal test results or history of head trauma with loss of consciousness, pregnancy or lactation were excluded.

Patients were instructed and also agreed not to take any regular medications for the treatment of migraines during observation period. In cases of severe pain, ibuprofen (300 mg each capsule with sustained release) was allowed as rescue medication during observation period. All migraine patients in this study were migraine-free for at least 72 h at the time of the MRI scan.

### Clinic variables measures

The demographic information (including age, gender, weight and height) of the participants were recorded. The clinical outcomes were the headache intensity (using a 0–10 visual analogue scale (VAS), 0 indicates no pain, 10 indicates worst pain ever) and the frequency of migraine attacks (the number of migraines separated by pain free intervals of at least 48 h of headache) based on patients’ headache diary according to the guidelines of the IHS for Clinical Trials in Migraine [24]. In addition, self-rating anxiety scale (SAS) and self-rating depression scale (SDS) were applied to assess the anxiety and depression status in MwoA patients [2, 25, 26].

### MRI data acquisition

MRI data was acquired on a 3.0 T Siemens Trio Tim system equipped with an 8-channel phase-array head coil at the West China Hospital MRI center. Prior to the functional run, a high-resolution structural image for each subject was acquired using a three-dimensional MRI sequence with a voxel size of 1mm^3^ employing an axial fast spoiled gradient recalled sequence (TR = 1900 ms; TE = 2.26 ms; data matrix, 256 × 256; field of view, 256 × 256 mm^2^). The blood oxygen level dependent (BOLD) resting-state functional images were obtained with echo-planar imaging (30 contiguous slices with a slice thickness of 5 mm; TR = 2000 ms; TE = 30 ms; flip angle, 90°; field of view, 240 × 240 mm^2^; data matrix, 64 × 64; total volumes, 180). All the participants were instructed to stay awake and to keep their heads still during the scan, with their eyes closed and ears plugged.

### Data analysis

#### Clinical data analysis

The baseline demographic information was analyzed using SPSS16.0 (SPSS Inc., Chicago, IL). Between-group comparisons were performed using two sample t-tests or Χ^2^, as appropriate. The significant level used for the statistical analysis with two sample t-tests was 5%. Continuous variables were presented as the mean with 95% confidence intervals (CI). Categorical variables were described as n (percentage).

#### VBM analysis

Pre-processing of structural images for VBM analyses was performed using SPM12 (www.fil.ion.ucl.ac.uk), and the CAT12 toolbox of the MatLab environment [21]. The images acquired for each participant were reoriented to have the same point of origin (anterior commissure) and spatial orientation. A non-linear deformation field was estimated that best overlaid the tissue probability maps on the individual subjects’ images. Three tissue components, including the gray matter (GM), white matter (WM), and cerebral spinal fluid (CSF), were obtained to calculate the overall tissue volume (GM, WM, and CSF volume) and total intracranial volume in the native space. Afterwards, all of the native-space tissue segments were registered to the standard Montreal Neurological Institute template (the standard included in SPM12) using the affine registration algorithm. The diffeomorphic anatomical registration through the exponentiated lie algebra (DARTEL) toolbox was applied to all participants’ GM and WM to refine the inter-subject registration. In the last step of DARTEL, the GM tissues are modulated using a non-linear deformation approach to compare the relative GM volume adjusted for individual brain size. Furthermore, the voxel values in the tissue maps are modulated by the Jacobian determinant that was calculated during spatial normalization [27]. Lastly, each participant’s modulated and normalized GM tissue segments were smoothed with an 8-mm full width at half maximum Gaussian filter. This involved segmentation of raw T1-weighted images into gray matter maps using SPM12, then registration using the nonlinear DARTEL algorithm to Montreal Neurological Institute space and resampling with a 8 mm smoothing kernel. Each tissue class (ie, GM) was processed independently after segmentation. After completing these image analyses, we obtained smoothed and modulated gray matter images to be used for statistical analysis. Two-sample t tests were used to compare the patients and healthy subjects, implementing the total intracranial volume as covariance. The significance of group differences was set at *P* < 0.05 using family-wise error correction.

#### Reho and seed based Functional Connectivity (FC) analysis

The rs-fMRI data preprocessing, Reho analysis and seed based FC analysis were performed using the Data Processing Assistant for Resting-State fMRI (DPARSF) software (available at:http://rfmri.org/DPARSF) in MatLab environment. The first 10 volumes were not analyzed to allow for signal equilibration effects. The fMRI images were slice timing corrected, head motion corrected, coregistered to respective structural images for each subject, segmented, regressed out of 6 head motion parameters, white matter signal and cerebrospinal fluid (CSF) signal, normalized by using structural image unified segmentation, and then re-sampled to 3-mm cubic voxels. We removed frames with FD > 0.5 mm (‘scrubbing’), one time point before ‘bad’ time points and two time points after ‘bad’ time points were deleted. The data was then detrended, bandpass filtered from 0.01 to 0.08 Hz and smoothed with a 8-mm full-width half-maximum (FWHM) Gaussian kernel for FC analysis.

##### Reho analysis

We compared the ReHo difference between MwoA patients and HS in SPM12. Individual Reho maps were generated by calculating Kendall’s coefficient concordance (KCC, also called ReHo value) of the time series of a given voxel with its nearest neighbors (26 voxels), on a voxel-wise basis [22]. Then, the data were smoothed with a Gaussian filter of 8 mm full width at half-maximum (FWHM) to reduce noise and residual differences in gyral anatomy. The ReHo maps were generated for each subject in each group. The significance of group differences was set at *P* < 0.05 using family-wise error correction for statistical analysis.

In order to find the possible regions with both structural (VBM) and functional (Reho) alterations in MwoA patients, the abnormal Reho and VBM maps of MwoA patients were overlapped. Only a region in the brainstem, named MRN [28], showed overlap in MwoA patients (Fig. 2 and Fig. 3). We then extracted the VBM and Reho values of the overlapped region in MRN in MwoA patients and healthy subjects. Pearson correlation analysis was applied between VBM or Reho values and headache intensity, headache frequency or disease duration of MwoA patients.

##### FC analysis

To further assess the possible correlation of the overlapped region in MRN with other brain regions in MwoA patients, resting state functional connectivity analysis was carried out by using the region in MRN as a seed. The averaged time course was obtained from the seed and the correlation analysis was performed in a voxel-wise way. Contrast images were generated for each subject by estimating the regression coefficient between all brain voxels and each seed’s time series, respectively. The correlation coefficient map was then converted into a Fisher-Z map by Fisher’s r-to-z transform to improve the normality. We compared the resting state functional connectivity difference between MwoA patients and healthy controls using two sample t-tests. A *P* < 0.05 family wise error corrected at cluster level was applied for all the comparisons.

## Results

### Baseline characteristics

There was no statistical difference between the MwoA patients and the healthy subjects, in age, gender, weight and height (*P* > 0.05) (Table 1).

**Table 1:** Baseline characteristics of MwoA patients and healthy subjects

Characteristics	MwoA, ***n*** = 72	HS, ***n*** = 46	***P*** value*
**Female n(%)**	57 (79.2%)	34 (79.1%)	0.907
**Age** (y) **Mean (95%CI)**	21.30 (20.89–21.73)	21.24 (20.98–21.50)	0.789
**Height** (cm) **Mean (95%CI)**	160.22 (158.49–161.96)	161.11 (158.49–161.96)	0.493
**Weight** (kg) **Mean (95%CI)**	52.49 (50.56–54.42)	51.13 (49.33–52.93)	0.335
**Duration** (mo) **Mean (95%CI)**	66.75 (32.19–101.31)	–	–
**Headache intensity Mean (95%CI)**	5.55 (4.41–6.69)	–	–
**Headache frequency Mean (95%CI)**	5.89 (2.62–9.16)	–	–
**SAS score Mean (95%CI)**	46.11 (37.17–55.06)	–	–
**SDS score Mean (95%CI)**	45.73 (35.51–55.95)	–	–

*HS* healthy subjects, *MwoA* migraine without aura, *SAS* self-rating anxiety scale, *SDS* self-rating depression scale

*, χ^2^ test was applied for gender comparison, two-sample t test was applied for the rest comparisions, between MwoA patients and healthy subjects

### VBM and Reho results

#### VBM

**Fig. 1:**
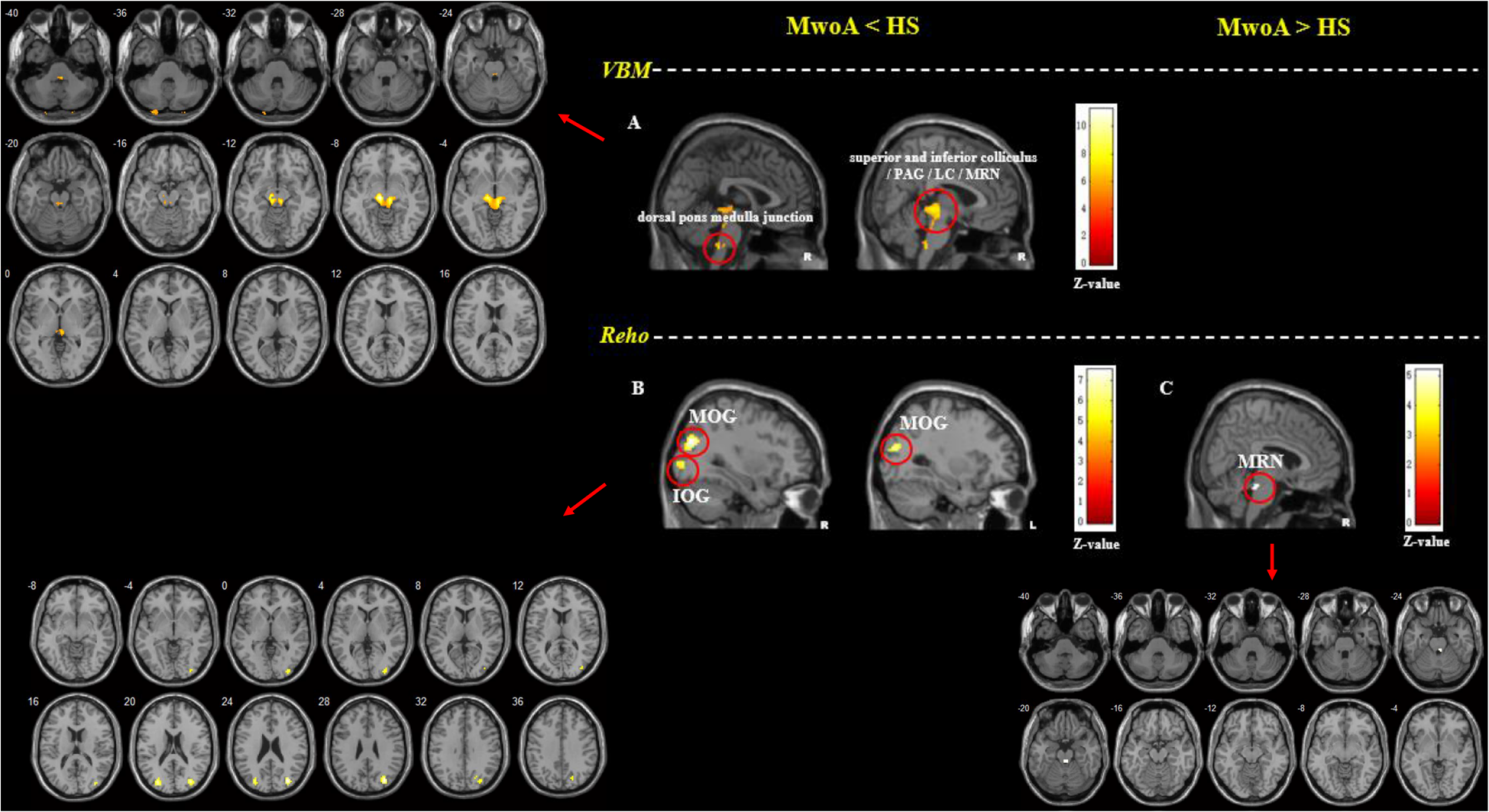
The VBM and Reho comparisons between MwoA patients and healthy subjects. **a**. MwoA patients showed lower gray matter volumes in the bilateral superior and inferior colliculus, PAG, LC, MRN and dorsal pons medulla junction compared to HS; **b**. MwoA patients showed lower Reho values in the right MOG and bilateral IOG compared to HS; **c**. MwoA patients showed higher Reho values in bilateral MRN compared to HS. A threshold of *P* < 0.05 family wise error (FWE) correction at cluster level were applied for all comparisons. HS, healthy subjects; IOG, inferior occipital gyrus; L, left side; LC, locus ceruleus; MOG, middle occipital gyrus; MwoA, migraine without aura; PAG, periaqueductal gray; R, right side; Reho, regional homogeneity; MRN, median raphe nuclei; VBM, voxel-based morphometry

Compared with healthy subjects, MwoA patients showed decreased volume in the bilateral superior and inferior colliculus, periaqueductal grey (PAG), locus ceruleus (LC), median raphe nuclei (MRN) and dorsal pons medulla junction. MwoA patients showed no increased volume in any brain regions, compared with healthy subjects (Table 2 and Fig. 1).

**Table 2:** The VBM and Reho comparisons between MwoA patients and healthy subjects

Contrast	Voxels	Brain regions	peak MNI (x, y, z)			Z score
***VBM comparison between MwoA patients and healthy subjects***						
MwoA < HS	1508	bilateral superior and inferior colliculus / PAG / LC / MRN	-8	−23	−9	Inf
	109	bilateral dorsal pons medulla junction	3	−38	−44	6.78
MwoA > HS	No brain region above the threshold.					
***Reho comparison between MwoA patients and healthy subjects***						
MwoA < HS	95	right MOG	33	−75	27	6.79
	12	right IOG	21	−72	36	5.14
	36	left MOG	−30	−81	21	6.14
MwoA > HS	11	bilateral MRN	6	−33	−24	4.93

*HS* healthy subjects, *Inf* infinity, *IOG* inferior occipital gyrus, *LC* locus ceruleus, *MNI* Montreal Neurological Institute coordinate, *MOG* middle occipital gyrus, *MwoA* migraine without aura patient, *MRN* median raphe nuclei, *Reho* regional homogeneity, *VBM* voxel-based morphometry

A threshold of *P* < 0.05 family wise error (FWE) correction at cluster level were applied for all comparisons. Z score infinity in this table means the probability of the VBM value in the peak MNI(x = − 8, y = −23, z = −9) of healthy subjects > migraineurs is approximately 100%

#### Reho

Compared with healthy subjects, MwoA patients showed decreased Reho values in the right middle occipital gyrus, inferior occipital gyrus and left middle occipital gyrus, and increased Reho values in the bilateral MRN (Table 2 and Fig. 1).

**Fig. 2:**
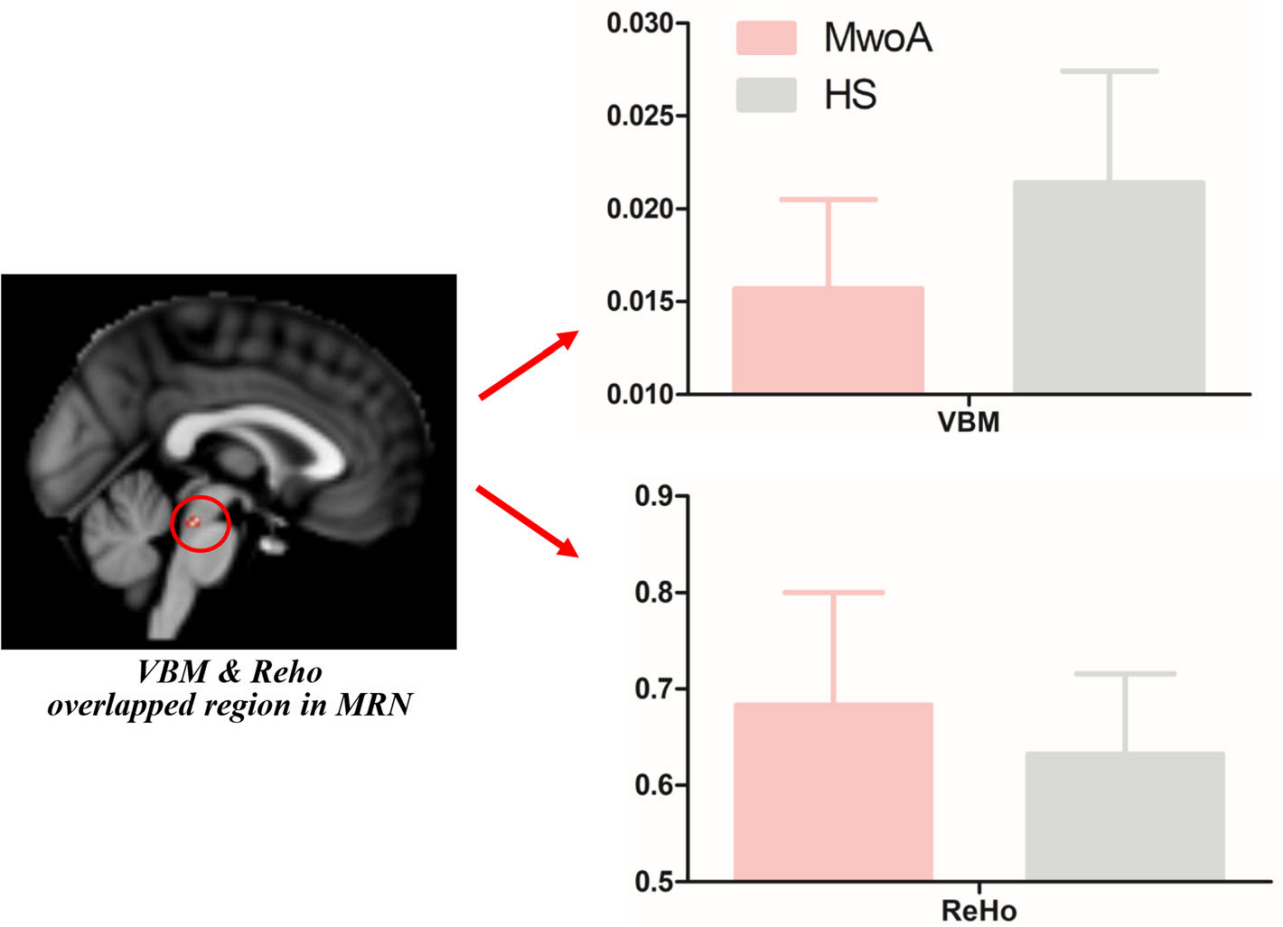
The VBM and Reho values of the overlapped region in MRN in MwoA patients and healthy subjects. MwoA had decreased gray matter volume but increased Reho values in the median raphe nuclei compared with healthy subjects. Pearson correlation analysis showed that there was no association between volume or Reho values of the MRN and headache frequency, headache intensity or disease duration, SAS and SDS in MwoA patients. HS, healthy subjects; MwoA, migraine without aura; Reho, regional homogeneity; MRN, median raphe nuclei; VBM, voxel-based morphometry

Only a region in the MRN [28] showed both structural (VBM) and functional (Reho) alterations in MwoA patients. MwoA had decreased volume but increased Reho values in the MRN compared with healthy subjects (Fig. 2). Pearson correlation analysis showed that there was no association between volume or Reho values of the MRN and headache frequency, headache intensity, disease duration, SAS or SDS in MwoA patients (VBM-headache frequency, *r* = 0.025, *p* = 0.837; VBM-headache intensity, *r* = − 0.029, *p* = 0.809; VBM-disease duration, *r* = 0.003, *p* = 0.977; VBM-SAS, *r* = − 0.039, *p* = 0.742; VBM-SDS, *r* = − 0.036, *p* = 0.762; Reho-headache frequency, *r* = 0.049, *p* = 0.682; Reho-headache intensity, *r* = − 0.023, *p* = 0.847; Reho-disease duration, *r* = 0.137, *p* = 0.250; Reho-SAS, *r* = 0.058, *p* = 0.628; Reho-SDS, *r* = 0.126, *p* = 0.290).

### Median raphe nuclei FC results

**Fig. 3:**
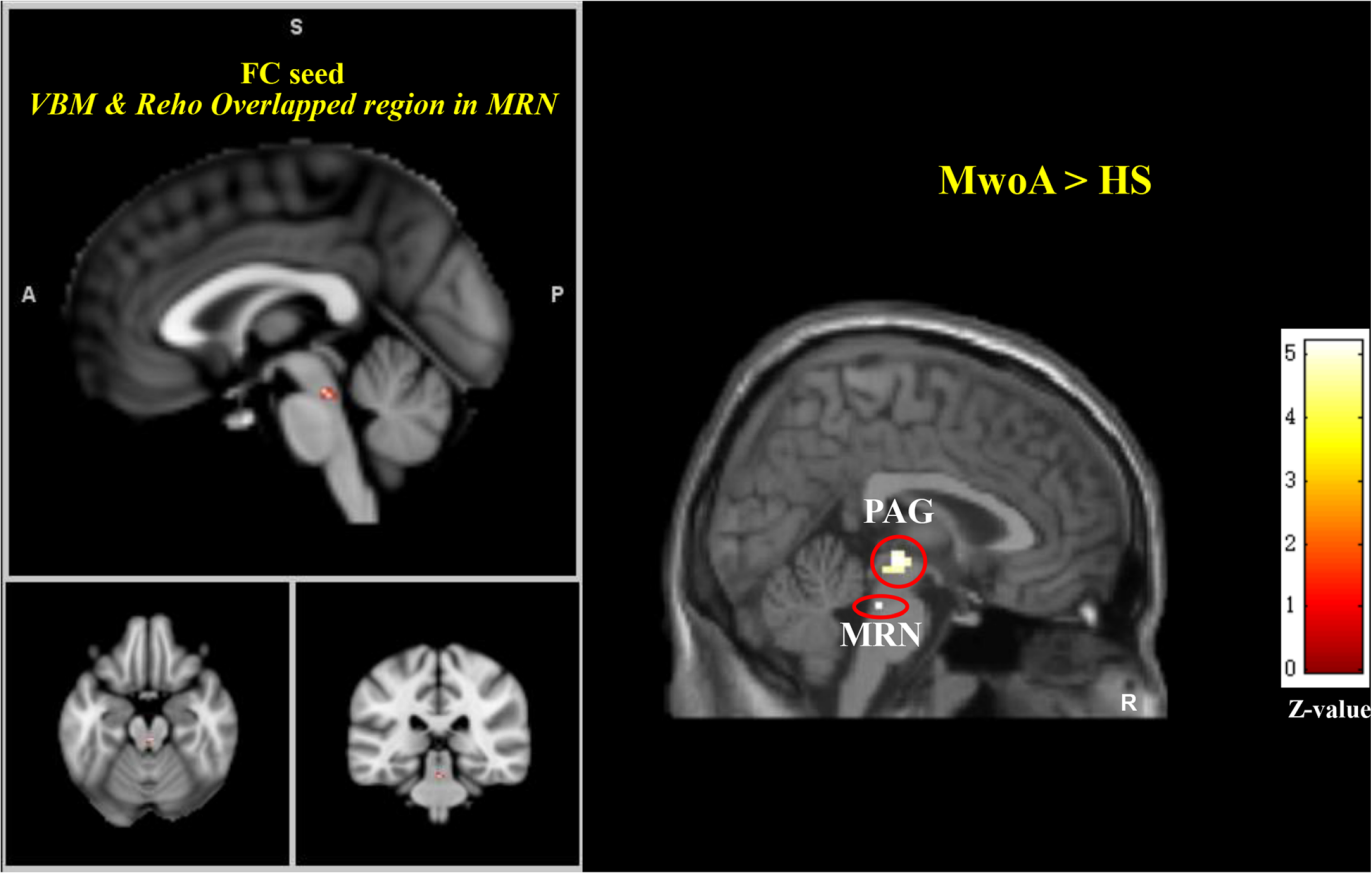
Altered median raphe nuclei functional connectivity in MwoA patients. MwoA patients showed higher median raphe nuclei functional connectivity with local median raphe nuclei and PAG compared to HS. A threshold of *P* < 0.05 family wise error (FWE) correction at cluster level were applied for all comparisons. A, anterior; FC, functional connectivity; HS, healthy subjects; MwoA, migraine without aura; P, posterior; PAG, periaqueductal gray; R, right side; Reho, regional homogeneity; MRN, median raphe nuclei; S, superior; VBM, voxel-based morphometry

Compared with healthy subjects, MwoA patients showed increased FC between the MRN and PAG. MwoA patients showed no decreased FC between the MRN and any other brain regions, compared with healthy subjects (Table 3 and Fig. 3).

**Table 3:** Altered median raphe nuclei functional connectivity in MwoA patients

Contrast	Voxels	Brain regions	MNI (x, y, z)			Z score
***Median raphe nuclei FC comparison between MwoA patients and healthy subjects***						
MwoA > HS	28	bilateral PAG	3	−21	−6	5.19
	5	bilateral MRN	3	−30	− 24	5.18
MwoA < HS	No brain region above the threshold.					

*HS* healthy subjects, *MNI* Montreal Neurological Institute coordinate, *MwoA* migraine without aura patient, *MRN* median raphe nuclei, *PAG* periaqueductal gray

A threshold of *P* < 0.05 family wise error (FWE) correction at cluster level were applied for all comparisons

We did not find any correlation between the MRN functional connectivity changes and patients’ clinical features as well as between the other volume or Reho alterations and patients’ clinical variables. To further test the reliability of these results, we added age and gender as covariates for re-analysis of Reho, VBM and FC and got similar results.

## Discussion

This study found that MwoA patients had decreased volume but increased ReHo in MRN. Besides, the MRN with both structural and functional alteration had increased resting state functional connectivity with PAG in MwoA patients. However, the correlation analysis showed that the VBM and Reho values of the MRN were not correlated with headache frequency, headache intensity, disease duration, SAS or SDS. This structural and functional neuroimaging study supports the involvement of MRN in the pathophysiology of migraine.

Regional homogeneity (Reho) is a method that applies Kendall’s coefficient of concordance to resting-state BOLD fMRI data in order to measure the similarity of the time series of a given voxel to those of its nearest neighbors in a voxel-wise way [22], which could reflect the local synchronization of spontaneous neural activity, hierarchical organization of the brain and neurodevelopmental factors [29]. Unlike functional connectivity or exploratory independent component analysis node-to-node connectivity which reflect interregional relationship between remote brain regions, Reho is a local spatial scale to measure functional interactions or synchronizations between the neighboring voxels or vertices [29]. Voxel-based morphometry (VBM) is a computational approach to neuroanatomy that measures differences in local concentrations of brain tissue, through a voxel-wise comparison of multiple brain images [21], which could reflect tissue atrophy or expansion [30]. Both Reho and VBM are data-driven analysis methods, reflecting the functional and structural status in vivo. Studies using Reho [31–34] or VBM [35–39] alone for patients with migraine have been reported. Whole-brain VBM or Reho studies identified widespread functional and volume alternations in migraineurs, specifically in the frontal cortex and limbic systems [31–39]. However, not all studies reported consistent, stable and replicable findings. To our knowledge, this is the first neuroimaging study combining Reho, VBM and FC to explore the concurrent functional and structural differences in migraineurs, which may provide specific and more reliable insights into the unfolding brain’s adaptive or maladaptive changes in migraineurs.

It is widely accepted that migraine involves activation and sensitization of trigeminovascular pathways [40], as well as brainstem and diencephalic nuclei [41]. This study found that MwoA patients had a concurrent brain structural and functional alteration in MRN. MRN is located in the brainstem, extending from the caudal edge of the superior cerebellar peduncles to the motor nucleus of the Vth cranial nerve [42]. Previous studies report that MRN plays a critical role in the regulation of hippocampal activity and is likely involved in memory consolidation processes [43], depression [44] and anxiety [45]. Besides, MRN is also an important brain region in the human ascending arousal system, which is related with consciousness maintenance and its disorders such as fatigue and sleep disturbance [28]. In migraine patients, recent transcranial sonography studies reported that a hypoechogenic MRN correlated to a higher migraine attack frequency [46] and depression [47]. MRN is the main source of 5-hydroxytryptamine (5-HT, also known as serotonin) in the brain [48]. Serotonin is a neurotransmitter that is affected by many physical and emotional processes, including depression, mood, social functioning, exercise, and diet [49]. In migraineurs, decreased levels of serotonin have been observed [50]. Serotonin receptors have been found on the trigeminal nerve and cranial vessels and their agonists especially triptans are effective in migraine treatment [51]. Although there is a growing body of evidence for a direct role for dysfunctions of central 5-HT and MRN availability in migraine, the exact and specific action of endogenous 5-HT system and MRN in migraine continues to be the focus of active investigation [46, 47, 52].

In order to further explore the MRN’s correlation with other brain regions in migraineurs, resting state functional connectivity analysis was applied by taking the region in MRN as the seed. This study found that the MRN had increased functional connectivity with PAG in migraineurs relative to HS. Previous animal neuroanatomy studies reported that the afferences for MRN mainly came from the limbic system, while the efferences were mainly to the lateral cortex, hypothalamus, amygdala, hippocampus, and medial cortex [42]. Some studies also report that MRN contribute serotoninergic projections to both the PAG (especially ventrolateral PAG) and the superior colliculus, the neural circus of which is related with a brain aversive system and pain modulation [53, 54]. The PAG plays a central role in descending pain modulatory system and is closely associated with opioid analgesia [55]. Animal studies also showed that descending modulation of the trigeminocervical complex (TCC), through the ventrolateral PAG and rostral ventromedial medulla, could cause the activation of ‘on’ cells and the inhibition of ‘off’ cells in the rostral ventromedial medulla, which seems to be critical for activation of TCC and development of migraine headache [41, 56, 57]. Taken together, this structural and functional neuroimaging study provide a more reliable evidence supporting the involvement of MRN in the pathophysiology of migraine.

There are several potential limitations in this study. 1) HS did not have SAS and SDS examined, which may have left the depression and anxiety level uncontrolled for migraineurs in this study. 2). This study primarily focused on the headache frequency, headache intensity and disease duration in migraineurs, with the symptoms such as allodynia, fatigue and sleep disturbances unrecorded. 3) This is a descriptive, not a mechanistic study. 4) The time elapsed between the MRI exam and the following migraine attack was not recorded, which might include the prodrome phases as the potential confounding bias. 5) The MRN structure and function alternations in this study are in alignment with many other neuroimaging studies. However, structural changes might affect the local non-linear registration fields and give rise to an observation of falsely positive functional MRI changes. This is a potential limitation for this study.

## Conclusion

Concurrent brain structural and functional alterations in MRN suggest that this structure is involved in the pathophysiology of migraine during the interictal period, which might help to better understand the symptoms in migraineurs.

## Data Availability

Clinical, neurophysiological and statistical data will be available upon request from any qualified investigator.

## Abbreviations

fMRI: Functional Magnetic Resonance Imaging
MwoA: Migraine without aura
HS: Healthy subjects
VBM: Voxel-based morphometry
Reho: Regional homogeneity
FC: Functional Connectivity
MRN: Median Raphe Nuclei
PAG: Periaqueductal gray matter
VAS: Visual analogue scale
IHS: International Headache Society
SAS: Self-rating Anxiety Scale
SDS: Self-rating Depression Scale
BOLD: Blood oxygen level dependent
GM: Gray matter
WM: White matter
CSF: Cerebral spinal fluid
FWHM: Full-width half-maximum
KCC: Kendall’s coefficient concordance
PAG: Periaqueductal grey
LC: Locus ceruleus
5-HT: 5-hydroxytryptamine
TCC: Trigeminocervical complex
